# Abscess of the Breast

**Published:** 1848-01

**Authors:** 


*Abscess of the Breast.*—"Subcutaneous inflammation of the Breast proceeds much as an ordinary phlegmon. When the abscess is formed between the mamma and the chest, the swelling is considerable, the breast raised up, but after an incision the cure usually takes place rapidly. But when the phlegmasia invades the substance of the breast itself, it is rare to find only a single abscess produced. We sometimes see 10, 20, 40 or 50 manifesting themselves in succession. An instant's reflection will show that this result is a natural consequence of the anatomical disposition of the inflamed tissue. The glandular parenchyma consists of different lobules, each of which constitutes a little organ having its own function, and which may become heated and irritated under the influence of lactation. Each lobule does not attain at the same time the same degree of irritation. One first inflames, then suppurates, and constitutes a first abscess: a neighboring lobule then becomes affected and, in its turn, forms an abscess; and so it may go on with all of them until we have as many successive abscesses as there are lobules.

"This distinction of abscesses of the breast into at least three orders is of the highest importance; and if we do not adopt it, our ideas upon the subject will be but very vague, and devoid of all precision as respects prognosis and treatment. Parenchymatous abscesses may last four or six months, or a year even, according to the rapidity of their succession and their number. The subcutaneous abscess last only as long as an ordinary phlegmon; and the submammary abscess has not the long duration of the parenchymatous one.

"Each of these has again its special treatment. We may endeavor to procure the resolution of *subcutaneous abscess*, and that by ordinary means; and, if suppuration occurs, we open it promptly, in order to avoid the burrowing of the pus among the tissues. *Sub-mammary phlegmon* should be treated especially by general measures, and leeches around the nipple. Topical applications are of little use, as they are separated from the centre of inflammation by the whole substance of the mammary gland. When an abscess is formed here, its prompt evacuation is desirable: but the perception of fluctuation is difficult, for the pus is surrounded by a large mass of tissues, and the thoracic parietes have not fixity enough to serve as a point of support. Nevertheless, you may recognise the existence of pus by the following characters: 1. An acute phlegmon rarely exists more than seven or eight days without suppuration taking place. 2. The breast is raised up like a sponge, and if we press upon it, it seems as if it were lying on a bladder full of fluid. 3. We find the breast surrounded by a kind of inflammatory œdema. Having recognised the pus, we should let it out promptly, or we expose ourselves to seeing it traverse the gland and form one of those abscesses I call *shirt-buttons*, These abscesses, moreover, have a mischievous influence upon the chest, and may lead to a purulent pleurisy. They may, too penetrate into the cellular tissue for a distance, and give rise to a diffused phlegmon. The incision should be made into the most dependent part, the place of election being below and at the outer side of the nipple, but, in some cases, a projecting point of the abscess indicates the place at which the opening should be made. It is always advantageous to make the incision towards the circumference of the breast, because the gland itself is not touched, and its weight tends to expel the pus. The bistoury should be directed almost parallel with the thoracic parietes, so as to slide it in between these and the mamma. The danger of such incisions is not great, there being no large arteries to fear. *Parenchymatous phlegmon* requires an energetic and varied treatment. Bleeding, purging, and the socalled anti-lactal medicines When pus forms, which is almost always the case, topical applications and incisions seldom prevent the successive implication of the lobules. Nevertheless, there is some advantage derived from the prompt opening of the abscess, *if the patient agrees to it;* for you should recollect that, in practice, if you open one abscess and others form, she never fails attributing these to your proceedings. These details will, I think, suffice to show you how important it is to distinguish the different abscesses of the breast, and to explain to you the confusion which prevails in the minds of some surgeons as regards their treatment."—*Gazette des Hopitaux*, No. 89.

